# IL-17 is Aberrantly Overexpressed Among Under-treatment *Systemic Lupus Erythematosus* Patients

**DOI:** 10.30699/ijp.2019.94878.1934

**Published:** 2019-08-01

**Authors:** Saeed Mohammadi, Sima Sedighi, Ali Memarian

**Affiliations:** 1 Stem Cell Research Center, Deputy of Research and Technology, Golestan University of Medical Sciences, Gorgan, Iran; 2 Infectious Diseases Research Center, Golestan University of Medical Sciences, Gorgan, Iran; 3 Department of Rheumatology, Golestan Rheumatology Research Center, Deputy of Research and Technology, Golestan University of Medical Sciences, Gorgan, Iran; 4 Department of Medical Immunology, Golestan Research Center of Gastroenterology and Hepatology, Deputy of Research and Technology, Golestan University of Medical Sciences, Gorgan, Iran

**Keywords:** Systemic lupus erythematosus, IL-17, glucocorticoids, pathogenesis, organ damage, treatment

## Abstract

**Background & Objective:**

*Systemic lupus erythematosus* (SLE) is an autoimmune disease with chronic inflammatory immune response. Current therapies mostly rely on *glucocorticoids* which are accompanied by side-effects and mostly fail to achieve a favorable remission. Th17 subpopulation of T cells is increased in exacerbated SLE as IL-17 cytokine is overexpressed. However, IL-17 is reported to be resistant to *glucocorticoids *in various disorders. Here, we evaluated the plasma level of IL-17 among newly diagnosed and under-treatment SLE patients to understand the effect of *glucocorticoids* on Th17 response.

**Methods:**

A total of 40 female SLE patients and 20 age- and sex- matched normal subjects were enrolled. IL-17 plasma level was evaluated using ELISA cytokine assay and analyzed with previously obtained IL-10, IFN-γ, and GILZ levels.

**Results:**

Our findings revealed that IL-17 was overexpressed among under-treatment SLE patients. There was a significant correlation between IL-17 and IFN-γ and significant reverse correlations between IL-17, IL-10, and GILZ levels. IL-17 was not significantly correlated with the disease activity.

**Conclusion:**

According to the role of IL-17 in tissue injury and the fact that *glucocorticoids* are not successful in preventing organ damages in SLE, the overexpressed IL-17 in response to therapies could be introduced as an underlying reason.

## Introduction

*Systemic lupus erythematosus* (SLE) is an autoimmune disorder of unknown etiology in which the chronic inflammatory immune response results in diverse clinical symptoms (1). Similar to other inflammatory and autoimmune disorders, various immunosuppressive treatments including antimalarial drugs and *glucoco-rticoids* are often prescribed to manage SLE. However, routine approaches have remained partially unsuccessful in inducing remission (2). Therefore, clinicians tend to employ more aggressive therapies including high doses of *glucocorticoids* which are not only accompanied by irreversible side-effects but also fail to prevent end-organ damages in some cases (3, 4). 

The role of T cell subtypes in the pathogenicity of SLE is established. T cells are capable of mediating immune response and/or tolerance upon maturation and activation. Therefore, the persistent inflammation in SLE could be attributed to the aberrant phenotypic alterations of T cells (5). A successful treatment should be capable of retrieving the lost balance between inflammatory T helper cell subtypes (Th1 and Th17) and regulatory T cells (Treg) to induce an authentic remission (6). Th1 cells and their mediator cytokines including interferon gamma (IFN-γ) are involved in cell-mediated immune response (7), while Th17 cells are contributed to tissue injury and organ damage by secreting IL-17 pro-inflammatory cytokines (8). Accordingly, Th17 subpopulation of T cells is reported to be increased in exacerbated SLE as IL-17 cytokine is overexpressed (9). 

It has been reported that IL-17 could be resistant to the regulation of *glucocorticoids* in various diseases such as asthma and Crohn’s disease (10). Recent findings have revealed that IL-17 cytokine family comprises of different ligands including IL-17 (IL-17A; CTLA-8), IL-17B, IL-17C, IL-17D, IL-17E (IL-25), and IL-17F, which are not only produced by T cells but other tissues such as prostate and fetal kidney (11). Each of these ligands activate their specific receptors in a multimeric manner and exert several immunoregulatory effects on other cell types by affecting the expression of several other cytokines and growth factors (12). Due to the wide signature of Th17 target genes, IL-17 could be involved in enormous physiological and pathologic processes such as tissue remodeling, acute phase response, anti-microbial activities, and also the pathology of autoimmunity (13, 14). Moreover, there are existing evidences in favor of the aberrant Th17/Th1 imbalance in SLE which is linked to the administration of *glucocorticoids *(15). However, there is still a lack of evidence explaining how *glucocorticoids*, even in extremely high doses, are not capable of inducing complete remission and end organ damage prevention in SLE. According to the remarkable role of Th17 response in tissue injury, IL17 level and TH17/Th1 balance could be introduced to be involved.

In the present study, we evaluated the plasma level of IL-17 among newly diagnosed and under-treatment SLE patients in correlation with the disease activity to elucidate the effect of glucocorticoids on Th17 response.

## Materials and Methods

Sample collection

We recruited 40 female SLE patients (18 under-treatment and 22 newly diagnosed) fulfilling 4 out of 11 items of the revised American College of Rheumatology (ACR) criteria (16) at *Sayyad Shirazi* educational hospital, rheumatology department, Golestan University of Medical Sciences, Gorgan, Iran. Patients with active infections, pregnancy, and/or history of other autoimmune diseases were not included in this study. Systemic Lupus Erythematosus Disease Activity Index (SLEDAI-2K) was used to calculate the disease activity by an expert rheumatologist (17). Twenty age- and sex- matched healthy subjects were also enrolled in the present case-control study. An informed consent letter was taken and signed by all participants following the Declaration of Helsinki (18). Whole blood samples were taken from all participants and plasma was separated as previously described (19) and stored at -80ºC until the measurement of IL-17. Clinical and laboratory data were obtained according to the registered files and a parallel research study (19).

ELISA cytokine assay

The commercially available ELISA kit (Biolegend, CA, USA) was used to determine the plasma level of IL-17 among SLE patients and healthy subjects following the manufacturer's protocol. The optical density of each sample was obtained at the wavelength of 450nm using Biotek ELISA reader ELX800 (Biotek, VT, USA). All samples were measured in triplicates and the results were reported as picograms per mL (pg/mL). 

Statistical Analysis

In order to analyze data statistically and prepare graphs, SPSS 22.0 and Graphpad Prism 5.04 software were used. Shapiro-Wilk test was conducted to address the normal distribution of variables in each group. All data were demonstrated as means±SE (standard Error). Kruskal-Wallis with Dunn-Bonferroni *post hoc* test was used to compare the means of multiple samples. Significant differences were also assessed using Mann-Whitney U test for comparing two independent samples. In order to evaluate the correlation between variables, spearman correlation study (two-tailed) was conducted. *P*-values lower than 0.05 were considered as statistically significant.

## Results

Clinical and Laboratory Findings

Our findings revealed that IL-17 plasma levels were significantly higher among patients receiving high doses of *glucocorticoids* (*P*=0.048). Moreover, we observed that IL-17 was significantly overexpressed among patients suffering from hair loss (*P*=0.043). Although mean concentration of IL-17 was higher among patients with lupus nephritis and malar rash, no significant difference was observed (Table 1). Correlation analyses between clinical and laboratory parameters with IL-17 plasma levels did not show any significant association (Table 2).

**Table1:** IL-17 expression within clinical characteristics of systemic lupus erythematosus patients

Characteristics (n=40)	IL-17 plasma level (pg/mL) (means±SE)			
	*Positive*	*n*	*Negative*	*n*
*Hair loss*	3.27±0.64	15	1.87±0.12	25
	**P-value=0.043** *			
*Lupus nephritis*	2.88±0.49	6	1.99±0.15	34
	**P-value=0.217**			
*Malar rash*	3.08±0.71	21	2.44±0.48	19
	**P-value=0.592**			
*High doses of GCs*	5.01±1.76	7	2.27±0.31	33
	**P-value=0.049**			

* P-values lower than 0.05 were considered as statistically significant. GCs: glucocorticoids.

**Table 2:** Correlation analysis of IL-17 plasma levels with clinical and laboratory characteristics

Characteristics*	IL-17 plasma level (pg/mL) (means±SE)	
	*r*_s_	P*-value*
*Anti-dsDNA titer (μM)*	-0.1926	0.2339
*WBC count (per μL)*	0.1963	0.2247
*RF-IgG (μM)*	-0.2832	0.0766
*ESR*	-0.1630	0.3148

* P-values lower than 0.05 were considered as statistically significant. Spearman correlation coefficient=rs, WBC: White Blood Cells, ESR: Erythrocyte Sedimentation Rate, RF: Rheumatoid Factor

IL-17 is overexpressed among under-treatment SLE patients

**Figure 1:**
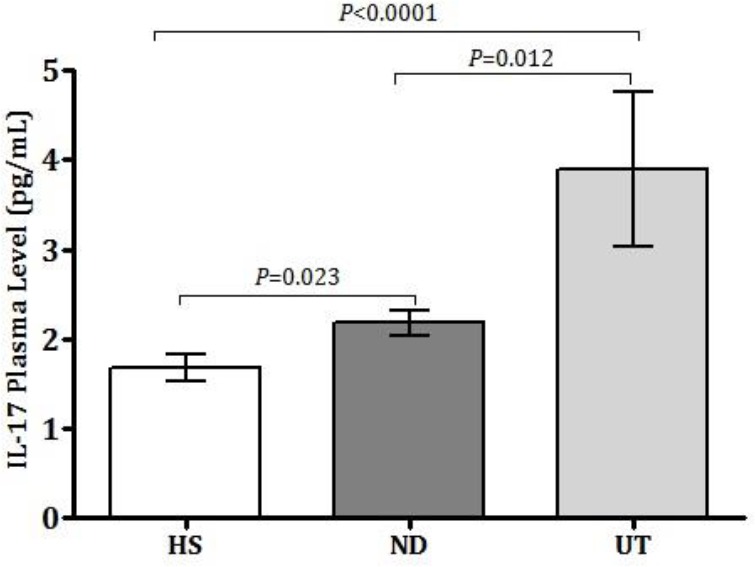
IL-17 plasma levels among SLE patients and healthy subjects; All experiments were repeated in triplicates for each sample. Data of each bar demonstrates means±SE. P-values lower than 0.05 were considered as statistically significant. SE: standard error; UT: Under treatment, ND: newly diagnosed, HS: healthy subjects

**Figure 2:**
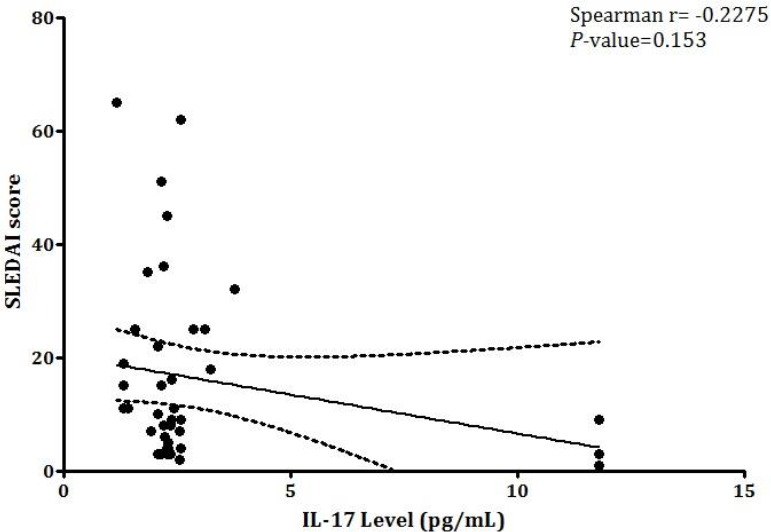
The correlation between IL-17 expression and SLEDAI-2K score; (Spearman correlation coefficient (rs) =-0.2275, P= 0.153). Two-tailed spearman correlation study was conducted to evaluate the correlation between IL-17 expression and SLEDAI-2K scores. P-values lower than 0.05 were considered as statistically significant

ELISA method was used to evaluate the plasma levels of IL-17 among newly diagnosed and under-treatment SLE patients in comparison to healthy subjects. Results revealed that the IL-17 production among patients receiving treatments was significantly higher than newly diagnosed patients (*P*=0.012) and healthy subjects (*P*<0.0001). Moreover, IL-17 was markedly higher among newly diagnosed patients compared to healthy subjects (*P*= 0.023) (Figure 1). Although there was a weak negative correlation between IL-17 plasma levels and SLEDAI-2K score, it was not statistically significant (r = -0.2275, *P* = 0.153) (Figure 2)

**Correlation analyses between IFN-γ, IL-10, GILZ, and IL-17 expression levels**

**Figure 3:**
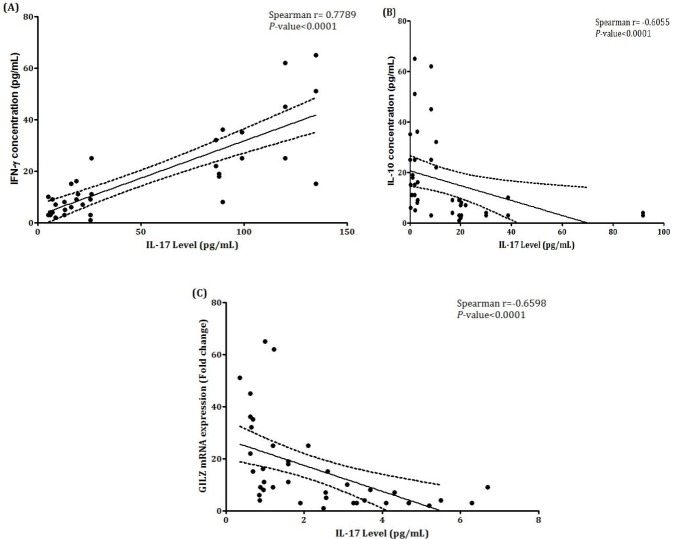
Correlation analyses of IL-17 plasma levels with IFN-γ, IL-10, and GILZ; (A) IL-17 was positively correlated with IFN-γ (rs = 0.7789, P<0.0001). (B) Relationship between IL-17 plasma expression and IL-10 level. IL-17 is negatively correlated with IL-10 (rs = -0.6055, P<0.0001). (C) Relationship between IL-17 and GILZ mRNA expression. IL-17 is negatively correlated with GILZ. (rs = -0.6598, P<0.0001). P-values lower than 0.05 were considered as statistically significant. (Note) Data of IFN-γ, IL-10, and GILZ have been presented in our previous study (19)

We obtained the expression data of IFN-γ, IL-10, and GILZ levels from previous studies on the same samples (19) and conducted a two-tailed spearman correlation study to evaluate the association with IL-17. As shown in Figure 3A, a significant positive correlation was seen between IL-17 and IFN-γ plasma levels (r_s_=0.7789, *P*<0.0001). There was also a significant reverse correlation between IL-17 and IL-10 plasma levels (r_s_=-0.6055, *P*<0.0001) (Figure 3B). As depicted in figure 3C, there was also a significant negative correlation between IL-17 plasma level and expression of GILZ mRNA (r_s_=-0.6598, *P*<0.0001).

## Discussion

SLE is an autoimmune disease in which the immune response is disturbed (1). The altered balance between cellular and humoral components of adaptive immune response and aberrant phenotypic changes of acting cells may finally result in a chronic inflammation and exert damages to multiple organs (20). T cell subtypes, including T helper cells (Th1 and Th17) and regulatory T cells (Treg) are of the major mediators in controlling inflammation with a dominant role in the pathogenicity of SLE. It has been suggested that Th17 cells are increased (21, 22) and Th17/Th1 response is dysregulated in SLE (23). Therefore, IL-17 production has a dominant role in the disease (24). Functional activity and frequency evaluation of Th17, Tc17, and other T-cell subsets has suggested disequilibrium of T-cell subsets in SLE which may attribute to the inflammatory response and disease pathogenesis (25). It is strongly believed that future therapies should target Th1, Th2, and Th17 profiles instead of inflammatory cytokines (26). A successful therapeutic approach should aim to recover the occurred imbalance in favor of a long-lasting remission and preventing end organ damages. However, current therapies including *glucocorticoids* and anti-malarial drugs mostly rely on eliminating clinical signs, managing the disease, and not achieving a favorable remission (1, 2). We previously demonstrated that *glucocorticoids* treatment exerts beneficial effects on Th1 response regulation by controlling the secretion of IFN-γ which also negatively correlated with the disease activity (19). Moreover, it is believed that glucocorticoid therapy is capable of skewing Th1 response toward immune regulation by decreasing the production of acting cytokines which is in favor of disease remission [19]. However, *glucocorticoids *may not be successful in modulating IL-17 production, especially in autoimmunity (10). 

Various research studies have claimed an increased IL-17 production in SLE patients and also significant correlation with the disease activity (21, 25, 27). In a recent study, Lopez et al. showed that the overexpression of IL-17 could be introduced as a pathogenic axis along with the overexpression of BLyS and IFNα (28). In addition, as Abdel Galil et al. claimed, IL-17 has been determined as a suitable biomarker of disease activity and remission predictor in SLE patients with nephritis (29). However, the SLE patients in the majority of these reports were not categorized on the basis of receiving *glucocorticoids*, while routine treatment approaches may have diverse effects on Th17 response. Although Zickert et al. (30) studied the association of IL-17 expression with response to treatment in SLE patients, receiving treatment was not a variable. Here, we evaluated the IL-17 plasma levels among *glucocorticoid*-receiving and newly diagnosed SLE patients in comparison to healthy subjects to delineate the alterations of IL-17 in response to routine *glucocorticoid* therapies. We observed that IL-17 was markedly higher among SLE patients receiving treatment in comparison to newly diagnosed patients and healthy subjects. Moreover, the mean concentration of IL-17 was significantly higher among patients receiving high doses of *glucocorticoids*. Although IL-17, as a pro-inflammatory cytokine, is overexpressed in SLE which is in accordance with previous reports (31), the under-treatment patients expressed higher levels of IL-17 which is not in favor of a proper remission. Regarding the dominant role of Th17 response in tissue damage (32), it could be claimed that the overexpressed IL-17 in response to *glucocorticoids* may be a possible reason why these therapeutic approaches are not as successful as expected. 

In order to better understand the IL-17 changes upon *glucocorticoid* therapy in inflammatory immune response of SLE, we evaluated the correlation of IL-17 plasma levels with IFN-γ and IL-10 expression levels, which were reported previously (19). There was a significant correlation between IL-17 and IFN-γ plasma levels and a reverse correlation between IL-17 and IL-10 as two major pro- and anti-inflammatory cytokines, which were in accordance with the findings by Zickert et al. (30). Moreover, we did not demonstrate any significant association between SLEDAI-2K score and IL-17. Although IL-17 had a significant correlation with IL-10 and IFN-γ cytokines, its level could not be a suitable indicator of disease activity. While these findings were not confirmed by Raymond et al. (33), they were supported by results of the study conducted by Vincent et al. (34). 

We have previously reported that *glucocorticoid-induced leucine zipper* (GILZ) was significantly overexpressed in response to treatment among SLE patients (19). GILZ is a transcription regulator which may play a dominant role in immune response through different ways (35). GILZ is also introduced as a molecule involved in differentiation of T cells, especially regulatory T lymphocytes (36). Here, we witnessed a negative correlation between IL-17 plasma level and GILZ mRNA expression. While GILZ was introduced as a molecule involved in mediating the anti-inflammatory properties of *glucocorticoids* (35, 37), the negative correlation of IL-17 and GILZ denotes a possible inflammatory and an unexpected effect of glucocorticoids on Th17 response. 

## Conclusion

IL-17 production has a major role in SLE pathogenesis, while Th17 cells are increased and Th17/Th1 response is dysregulated. Although administration of glucocorticoids exerts beneficial effects in disease management, its application has been controversial in achieving complete remission. Our findings showed that IL-17 was higher among under-treatment SLE patients compared to newly diagnosed and healthy subjects. The concentration of IL-17 was also higher among patients receiving high doses of *glucocorticoids*. According to role of IL-17 in tissue injury and the fact that glucocorticoids are not capable of preventing organ damages in SLE, the overexpressed IL-17 could be introduced as an underlying reason. However, further experiments should be conducted to confirm these findings.

## References

[1] Jemal Murphy G, Lisnevskaia L, Isenberg D (2013 ). Systemic lupus erythematosus and other autoimmune rheumatic diseases: challenges to treatment. Lancet.

[2] Yildirim-Toruner C, Diamond B (2011 ). Current and Novel Therapeutics in Treatment of SLE. J Allergy Clin Immunol.

[3] Moghadam-Kia S, Werth VP (2010). Prevention and treatment of systemic glucocorticoid side effects. Int J Dermatol.

[4] Qayyum A, Nagy AAH (2008). Immuno-histological changes in lupus nephritis in female patients: a four-year study. Saudi J Kidney Dis Transpl.

[5] Mak A, Kow NY (2014). The Pathology of T Cells in Systemic Lupus Erythematosus. J Immunol Res.

[6] Grondal G, Gunnarsson I, Ronnelid J, Rogberg S, Klareskog L, Lundberg I (2000). Cytokine production, serum levels and disease activity in systemic lupus erythematosus. Clin Exp Rheumatol.

[7] Theofilopoulos AN, Koundouris S, Kono DH, Lawson BR (2001). The role of IFN-gamma in systemic lupus erythematosus: a challenge to the Th1/Th2 paradigm in autoimmunity. Arthritis Res.

[8] Nalbandian A, Crispín JC, Tsokos GC (2009). Interleukin-17 and systemic lupus erythematosus: current concepts. Clin Exp Immunol.

[9] Pernis AB (2009 ). Th17 cells in rheumatoid arthritis and systemic lupus erythematosus. J Intern Med.

[10] Banuelos J, Shin S, Lu N (2015). Distinct glucocorticoid sensitivity of Th17 cytokines in murine T hybridomas and primary cells (IRC11P.428). J Immunol.

[11] Aggarwal S, Gurney AL (2002). IL‐17: prototype member of an emerging cytokine family. Journal of leukocyte biology.

[12] Shen F, Gaffen SL (2008). Structure-function relationships in the IL-17 receptor: implications for signal transduction and therapy. Cytokine.

[13] Yao Z, Painter SL, Fanslow WC, Ulrich D, Macduff BM, Spriggs MK (1995). Human IL-17: a novel cytokine derived from T cells. The Journal of Immunology.

[14] Yu JJ, Gaffen SL (2008). Interleukin-17: a novel inflammatory cytokine that bridges innate and adaptive immunity. Front Biosci.

[15] Prado C, de Paz B, Gomez J, Lopez P, Rodriguez-Carrio J, Suarez A (2011). Glucocorticoids enhance Th17/Th1 imbalance and signal transducer and activator of transcription 3 expression in systemic lupus erythematosus patients. Rheumatology.

[16] Hochberg MC (1997). Updating the American College of Rheumatology revised criteria for the classification of systemic lupus erythematosus. Arthritis Rheumatol.

[17] Touma Z, Urowitz MB, Gladman DD (2013). Systemic lupus erythematosus disease activity index 2000 responder index-50 website. J Rheumatol.

[18] General Assembly of the World Medical A (2014). World Medical Association Declaration of Helsinki: ethical principles for medical research involving human subjects. J Am Coll Dent.

[19] Mohammadi S, Ebadpour MR, Sedighi S, Saeedi M, Memarian A (2017 ). Glucocorticoid-induced leucine zipper expression is associated with response to treatment and immunoregulation in systemic lupus erythematosus. Clin Rheumatol.

[20] Solati K, Mousavi M (2015). The efficacy of mindfulness-based cognitive therapy on general health in patients with systemic lupus erythematosus: A randomized controlled trial. Journal of Kerman University of Medical Sciences.

[21] Yang J, Chu Y, Yang X, Gao D, Zhu L, Yang X (2009). Th17 and natural Treg cell population dynamics in systemic lupus erythematosus. Arthritis Rheum.

[22] El-Gwad ERA, Elshabacy FA, Abdul-Hafeez NA, Ameen SG (2016). Expression of intracellular interleukin-17 in systemic lupus erythematosus patients. Benha Med J.

[23] Shah K, Lee WW, Lee SH, Kim SH, Kang SW, Craft J (2010). Dysregulated balance of Th17 and Th1 cells in systemic lupus erythematosus. Arthritis Res Ther.

[24] Pan Q, Gong L, Xiao H, Feng Y, Li L, Deng Z (2017). Basophil Activation-Dependent Autoantibody and Interleukin-17 Production Exacerbate Systemic Lupus Erythematosus. Front Immunol.

[25] Henriques A, Ines L, Couto M, Pedreiro S, Santos C, Magalhaes M (2010). Frequency and functional activity of Th17, Tc17 and other T-cell subsets in Systemic Lupus Erythematosus. Cell Immunol.

[26] Guimaraes PM, Scavuzzi BM, Stadtlober NP, Franchi Santos L, Lozovoy MAB, Iriyoda TMV (2017 ). Cytokines in systemic lupus erythematosus: far beyond Th1/Th2 dualism lupus: cytokine profiles. Immunol Cell Biol.

[27] Wong C, Ho CY, Li E, Lam C (2000). Elevation of proinflammatory cytokine (IL-18, IL-17, IL-12) and Th2 cytokine (IL-4) concentrations in patients with systemic lupus erythematosus. Lupus.

[28] López P, Rodríguez-Carrio J, Caminal-Montero L, Mozo L, Suárez A (2016). A pathogenic IFNα, BLyS and IL-17 axis in Systemic Lupus Erythematosus patients. Scientific reports.

[29] Galil SMA, Ezzeldin N, El-Boshy ME (2015). The role of serum IL-17 and IL-6 as biomarkers of disease activity and predictors of remission in patients with lupus nephritis. Cytokine.

[30] Zickert A, Amoudruz P, Sundström Y, Rönnelid J, Malmström V, Gunnarsson I (2015). IL-17 and IL-23 in lupus nephritis-association to histopathology and response to treatment. BMC immunology.

[31] Raymond W, Ostli-Eilertsen G, Griffiths S, Nossent J (2017). IL-17A levels in systemic lupus erythematosus associated with inflammatory markers and lower rates of malignancy and heart damage: Evidence for a dual role. Eur J Rheumatol.

[32] Steinman L (2007). A brief history of TH17, the first major revision in the TH1/TH2 hypothesis of T cell-mediated tissue damage. Nat Med.

[33] Raymond W, Ostli-Eilertsen G, Griffiths S, Nossent J (2017). IL-17A levels in systemic lupus erythematosus associated with inflammatory markers and lower rates of malignancy and heart damage: evidence for a dual role. European journal of rheumatology.

[34] Vincent FB, Northcott M, Hoi A, Mackay F, Morand EF (2013). Clinical associations of serum interleukin-17 in systemic lupus erythematosus. Arthritis research & therapy.

[35] Beaulieu E, Morand EF (2011). Role of GILZ in immune regulation, glucocorticoid actions and rheumatoid arthritis. Nat Rev Rheumatol.

[36] Jones SA, Perera DN, Fan H, Russ BE, Harris J, Morand EF (2015 ). GILZ regulates Th17 responses and restrains IL-17-mediated skin inflammation. Journal of autoimmunity.

[37] Hu X, Li WP, Meng C, Ivashkiv LB (2003 ). Inhibition of IFN-gamma signaling by glucocorticoids. J Immunol.

